# Supplementary material for: Anatomy of a failure: a retrospective evaluation of a cognitive bias modification intervention to promote physical activity in cardiac rehabilitation
Source: BMJ Open. 2026 Apr 29;16(4):e101558. doi: 10.1136/bmjopen-2025-101558 (PMC13141020; doi:10.1136/bmjopen-2025-101558)
Supplement: Supplementary file 2 [file bmjopen-2025-101558.reviewer_comments.pdf]

PEER REVIEW HISTORY

BMJ Open publishes all reviews undertaken for accepted manuscripts. Reviewers are asked to complete a checklist review form and are provided with free text boxes to elaborate on their assessment. These free text comments are reproduced below.

ARTICLE DETAILS

Title (Provisional)

Anatomy of a Failure: A Retrospective Evaluation of a Cognitive Bias Modification Intervention to Promote Physical Activity in Cardiac Rehabilitation

Authors

Fessler, Layan; Maltagliati, Silvio; Meyer, Philippe; Finckh, Axel; Cullati, Stéphane; Sander, David; Friese, Malte; Wiers, Reinout W.; Farajzadeh, Ata; Luthy, Christophe; Sarrazin, Philippe; Cheval, Boris

---

VERSION 1 - REVIEW

**Reviewer**	**1**
**Name**	**Rechcinski, Tomasz**
**Affiliation**	**Medical University of Lodz**
**Date**	**04-Jun-2025**
**COI**	**None**

I congratulate the authors on their decision to publish the results of the study that ended in failure (answer "No" in p. 3 and 9). Despite such an outcome, reading this manuscript is very instructive, especially since the authors identified the reasons for the failure not only on the side of the patients, but also on the side of the staff and, in a sense, the way in which the study was organized. The authors proved that the path to constructing a good study methodology (especially when it comes to modifying human behavior) is difficult. The topic discussed is important - many risk factors for atherosclerosis can be modified pharmacologically, and low physical activity is one of the exceptions here. I wonder if the authors had access to information on the frequency of diagnosis of depression, kinesiophobia or hypothyroidism (including subclinical) - this could enrich the clinical characteristics of the study group. I also did not find information on the role of family members or friends in supporting patients in physical activity. In the country where I work with similar patients, cardiac rehabilitation after a heart attack is voluntary and I see a large role for close people in supporting patients in changing their attitude towards physical activity. To my knowledge, in Switzerland, cardiac rehabilitation after coronary intervention

is an integral part of treatment (in a sense mandatory), could the authors comment on this difference in approach as part of the discussion?

**Reviewer**	**2**
**Name**	**Hirashiki, Akihiro**
**Affiliation**	**Natinal Center for Geriatrics and Gerontology, Cardiology**
**Date**	**30-Aug-2025**
**COI**	**None**

This manuscript addresses an important and innovative topic: the feasibility and acceptance of a cognitive bias modification (CBM) intervention to enhance physical activity among cardiac rehabilitation patients. The study is timely, given this population's persistent difficulties in maintaining long-term adherence to physical activity. The manuscript is generally well written, and transparent in reporting the challenges and lessons learned. It provides valuable insights for researchers designing future interventions. However, several issues must be addressed to improve clarity and strengthen certain reporting aspects.

Major Strengths

1. The study tackles a highly relevant clinical and public health issue, promoting physical activity in cardiac rehabilitation.

2. The authors are transparent in reporting the limitations and the “negative” outcome, which adds to the literature on intervention feasibility.

3. Using behavioural, cognitive, and emotional acceptance indicators provides a multidimensional perspective.

Major Points

1. The abstract currently emphasises “failure.” While accurate, framing it more as “lessons learned” could make the message more constructive and appealing to a broader readership. Similarly, the conclusion could better highlight not only its limitations but also the positive contributions of the study (e.g., identification of barriers, guidance for future digital interventions).

Minor comments

1. In the Method and Results, the study population is unclear in this study. The authors should describe the study population clearly, including the inclusion and exclusion criteria, even though the previous study was described. Next, the Table needs to compare intervention and control groups, at least complete CBM and/or Complete PA, even though the patient numbers are small.

2, PA change from baseline to after intervention should be shown in the Table, even for small data.

3. In the Discussion, a comparison with other studies is recommended. The manuscript briefly notes attrition rates in CBM interventions for other health behaviours. This section could be expanded slightly to discuss whether the observed rates are typical for cardiac rehabilitation interventions more broadly, beyond CBM.

VERSION 1 - AUTHOR RESPONSE

Reviewer #1:

Dr. Tomasz Rechcinski, Medical University of Lodz

Comments to the Author:

I congratulate the authors on their decision to publish the results of the study that ended in failure (answer "No" in p. 3 and 9). Despite such an outcome, reading this manuscript is very instructive, especially since the authors identified the reasons for the failure not only on the side of the patients, but also on the side of the staff and, in a sense, the way in which the study was organized. The authors proved that the path to constructing a good study methodology (especially when it comes to modifying human behavior) is difficult. The topic discussed is important - many risk factors for atherosclerosis can be modified pharmacologically, and low physical activity is one of the exceptions here.

Response: We sincerely thank Dr. Rechcinski for his thoughtful and encouraging comments. We particularly appreciate his recognition of the value in transparently reporting implementation failures – a practice that, while challenging, is essential for advancing the field and helping other researchers avoid similar pitfalls. We agree that identifying barriers at multiple levels (patient, staff, and organisational) was crucial to understanding why the intervention failed. As Dr Rechcinski notes, modifying health behaviours such as physical activity poses a unique challenge compared to pharmacological interventions for cardiovascular risk factors. This highlights the need for more rigorous, prospectively planned evaluation frameworks when testing behavioural interventions in real-world clinical settings. We hope that openly sharing our experience will contribute to the development of more robust implementation strategies for future physical activity promotion interventions in cardiac rehabilitation and encourage other researchers to report on the lessons they have learned from facing similar implementation challenges.

I wonder if the authors had access to information on the frequency of diagnosis of depression, kinesiophobia or hypothyroidism (including subclinical) - this could enrich the clinical characteristics of the study group.

Response: Thank you for this valuable suggestion to enrich the clinical characterisation of our sample. Having reviewed the patients' medical records, we found that no formal clinical diagnoses of depression, kinesiophobia or hypothyroidism (including subclinical cases) had been documented at baseline. However, we did collect patient-reported outcome measures assessing psychological symptoms. As shown in Supplementary Table S1 and now reported in the main text (pages 9–10), patients self-reported low levels of anxiety and depressive symptoms, which is consistent with the absence of formal diagnoses.

Pages 9–10: "*Patients reported moderate levels of global physical health (M = 2.88, SD = 0.89, scale: 1–5), global mental health (M =3.77, SD = 0.98, scale: 1–5), and life satisfaction (M = 5.68, SD = 1.26, scale: 1–7). Patients also reported low-to-moderate levels of anxiety (M = 1.79, SD = 0.90, scale: 1–5), depressive symptoms (M = 1.62, SD = 0.76, scale: 1–5), fatigue (M = 2.55, SD = 1.06, scale: 1–5), and pain intensity (M = 2.10, SD, 2.29, scale: 1–5) in their daily life.*"

I also did not find information on the role of family members or friends in supporting patients in physical activity. In the country where I work with similar patients, cardiac rehabilitation after a heart attack is voluntary and I see a large role for close people in supporting patients in changing their attitude towards physical activity. To my knowledge, in Switzerland, cardiac rehabilitation after coronary intervention is an integral part of treatment (in a sense mandatory), could the authors comment on this difference in approach as part of the discussion?

Response: Thank you for raising this important point about family support, which we had not addressed properly. We also appreciate your insight into the differences between cardiac rehabilitation systems in different countries. In the Geneva cardiac rehabilitation programme, family members are invited to attend sessions, especially the introductory session and the 12 round-table discussions held during the programme. However, medical staff reported that only around 10% of patients were accompanied by a relative to at least one session. With regard to the mandatory nature of cardiac rehabilitation, while it is strongly recommended in Switzerland following a myocardial infarction, it is not legally mandatory.

We agree that these factors are highly relevant to our understanding of the results. At baseline, participants reported moderate intentions to engage in regular physical activity (M = 5.51, SD = 1.34 on a 0–10 scale; see Supplementary Table S1). The combination of modest baseline motivation, very limited family support and the voluntary nature of participation may have created an environment in which an additional cognitive intervention was perceived as burdensome rather than supportive and faced substantial engagement barriers. As you mentioned, family support plays a crucial role in facilitating behavioural change, and its near absence in our study is an important limitation. We have substantially revised the Discussion (pages 14–15) to explicitly address the following: (1) the role of limited family involvement as a barrier to engagement, (2) how these factors interacted with baseline motivation to influence programme uptake, and (3) the context of voluntary cardiac rehabilitation systems on motivation. We have also added family involvement to Table 1 as a modifiable barrier, providing concrete recommendations for future implementation.

Pages 14–15: "*Another cognitive indicator can be found in the reported moderate baseline intentions to engage in regular PA (M = 5.51, SD = 1.34 on a 0–10 scale; see Supplementary Table S1), which may have been insufficient to motivate engagement with an additional intervention specifically targeting PA behaviour. This modest motivation suggests that the programme may have been targeting patients who were not yet ready for such an intervention, or that the intervention itself failed to sufficiently enhance motivation. In addition, the lack of family involvement may have compounded these challenges further. The medical team reported that only approximately 10% of patients were accompanied by a family member during programme sessions, despite explicit invitations. Family involvement has been identified as a strong predictor of adherence to cardiac rehabilitation programmes,*[64] *and its near absence in our study likely further undermined engagement. Finally, although cardiac rehabilitation is strongly recommended following a myocardial infarction in Switzerland, participation is voluntary rather than mandatory. Therefore, the combination of modest baseline motivation, limited external support from family members, and the voluntary nature of cardiac rehabilitation participation may have created an environment where an additional cognitive intervention—already perceived as burdensome rather than supportive—faced significant engagement barriers.*"

Page 15: "*Future implementations should consider strategies to systematically involve family members in supporting patient engagement, assess baseline readiness for behaviour change*

interventions, and carefully consider how additional programmes are framed and integrated within existing rehabilitation structures."

Table 1. Potential Reasons for Failure and Recommendations for Future Studies

Issue	Reasons	Recommendations
Difficulty with patient enrolment	Protocol perceived as separate from care, high commitment burden.	Integrate the protocol as a central part of rehabilitation.
		Family support.
		Explore low-friction, automated interventions that minimise participant burden (e.g., Heartphone app[25]).
	Research assistants not fully integrated in the hospital unit.	Ensure research assistants are fully embedded in the healthcare team.
	Inconsistent research assistant availability.	Align training sessions with patient schedules to minimise perceived burden.
	Logistical issues with tablets.	Ensure regular maintenance for all equipment.
Difficulty with patient acceptance	Patients perceived the task as useless and boring.	Enhance the perceived value of the task by linking it clearly to PA behaviour and reducing its monotony.
	Task was lengthy, monotonous, repetitive and of low subjective value.	Including animations and "vitality gauges" that can increase or decrease depending on the action (e.g., approaching or avoiding PA).
	Lack of perceived connection between the task and its impact on PA behaviour.	Integrate more engaging and intrinsically motivating tasks, such as gamification or consequence-based interventions (e.g., ABC training[27]).
	Scepticism about the effectiveness of digital interventions.	Employ emerging technologies like virtual reality to create more appealing interventions.
		Co-production of protocol and pilot study.

Abbreviations: ABC, Antecedents Behaviours Consequences; PA, physical activity.

Reviewer #2:
Dr. Akihiro Hirashiki, Natinal Center for Geriatrics and Gerontology

Comments to the Author:

This manuscript addresses an important and innovative topic: the feasibility and acceptance of a cognitive bias modification (CBM) intervention to enhance physical activity among cardiac rehabilitation patients. The study is timely, given this population's persistent difficulties in maintaining long-term adherence to physical activity. The manuscript is generally well written, and transparent in reporting the challenges and lessons learned. It provides valuable insights for researchers designing future interventions. However, several issues must be addressed to improve clarity and strengthen certain reporting aspects.

Major Strengths

1. The study tackles a highly relevant clinical and public health issue, promoting physical activity in cardiac rehabilitation.

2. The authors are transparent in reporting the limitations and the "negative" outcome, which adds to the literature on intervention feasibility.

3. Using behavioural, cognitive, and emotional acceptance indicators provides a multidimensional perspective.

Response: We sincerely thank Dr. Hirashiki for his thorough and constructive review. We greatly appreciate his recognition of the value in transparently reporting implementation challenges and the multidimensional approach to assessing acceptability. We have carefully addressed all comments below and believe these revisions have substantially strengthened the manuscript's clarity and rigor.

Major Points

1. The abstract currently emphasises "failure." While accurate, framing it more as "lessons learned" could make the message more constructive and appealing to a broader readership. Similarly, the conclusion could better highlight not only its limitations but also the positive contributions of the study (e.g., identification of barriers, guidance for future digital interventions).

Response: We thank the reviewer for this valuable suggestion, which has helped us to achieve a more balanced approach. We have carefully revised both the Abstract and Conclusion to emphasise the constructive lessons learned and barriers identified, rather than focusing solely on negative outcomes. These changes make the message more actionable for designers of future interventions.

Regarding the title “Anatomy of a Failure”, we have deliberately retained this phrasing as we believe it serves an important purpose in scientific literature. Despite being crucial for advancing the field, publishing and clearly labelling “negative” results remains uncommon. It prevents wasted resources on similar unsuccessful approaches and normalises transparent reporting of implementation challenges. The provocative framing helps to ensure that this work reaches researchers who might otherwise overlook lessons from unsuccessful interventions. However, as the reviewer suggests, we have ensured that the abstract, body and conclusion strike a balance between this framing and constructive, forward-looking guidance. We believe that this approach — a candid title paired with constructive content — maximises both attention and utility for the cardiac rehabilitation research community.

Pages 2–3: “***Conclusion.*** *The IMPACT trial’s challenges in recruiting and retaining participants provide important lessons for designing and implementing future digital interventions to promote PA in cardiac patients. By systematically identifying barriers to engagement across patient, staff, and organisational levels, this study offers actionable guidance for improving the acceptability and feasibility CBM and similar digital behaviour change interventions in cardiac rehabilitation settings.*”

Pages 19–20: “*This study highlights both the challenges and opportunities in developing and implementing CBM-based PA interventions in cardiac rehabilitation. The barriers identified—including perceived task irrelevance, monotony, implementation burden, and limited family support—provide a roadmap for future intervention. Effective PA promotion requires approaches that are not only evidence-based but also acceptable, engaging, feasible, and seamlessly integrated into both patients’ lives and health care routines. By openly sharing these implementation lessons, we aim to accelerate the development of more effective digital interventions that can maximise the significant PA benefits of enhancing the health and quality of life of cardiovascular patients.”*

Minor comments

1. In the Method and Results, the study population is unclear in this study. The authors should describe the study population clearly, including the inclusion and exclusion criteria, even though the previous study was described.

<u>Response:</u> We thank the reviewer for this helpful comment. We agree that the study population needed clearer description. The Methods section has been revised to explicitly

state the inclusion and exclusion criteria for this cardiac rehabilitation cohort. The Results section and Supplementary Materials 4 now provide comprehensive demographic and clinical characteristics of the study population.

Page 5: "*Patients were eligible for inclusion if they were: (a) receiving treatment in an outpatient cardiac rehabilitation programme; (b) aged 18 years or older; (c) were physically and cognitively able to complete the study protocol; and (d) provided written informed consent to participate. Patients were excluded if they had a medical contraindication to physical. All eligibility criteria were verified by the medical team.*"

Pages 9–10: "*Descriptive characteristics of the sample are presented in Supplementary Material 1. A total of 68 outpatients enrolled in a cardiac rehabilitation programme were included in this evaluation. The majority were male (86.76%), with a mean age of 57.76 years (SD = 10.76). Mean left ventricular ejection fraction was 52.87% (SD = 10.49). The most common indication for cardiac rehabilitation programme was acute coronary syndrome, comprising 40.30% with non-ST-elevation myocardial infarction (NSTEMI) and 26.87% with ST-segment elevation myocardial infarction (STEMI). Nearly half of the participants (45.45%) were classified as overweight, and the majority reported engaging in some PA in daily life prior to programme enrolment. Patients reported moderate levels of global physical health (M = 2.88, SD = 0.89, scale: 1–5), global mental health (M =3.77, SD = 0.98, scale: 1–5), and life satisfaction (M = 5.68, SD = 1.26, scale: 1–7). Patients also reported low-to-moderate levels of anxiety (M = 1.79, SD = 0.90, scale: 1–5), depressive symptoms (M = 1.62, SD = 0.76, scale: 1–5), fatigue (M = 2.55, SD = 1.06, scale: 1–5), and pain intensity (M = 2.10, SD, 2.29, scale: 1–5) in their daily life.*"

Next, the Table needs to compare intervention and control groups, at least complete CBM and/or Complete PA, even though the patient numbers are small.
Response: Thank you for this important suggestion. We have now included comparative analyses between intervention and control groups for participants with complete physical activity data at programme end (n = 17; intervention: n = 10, control: n = 7). Given the very sample sizes are small and the fact that this study was not powered to detect between-group differences, we have placed these comparisons in Supplementary Material 2 (Tables S2–S3) with appropriate caveats, rather than in the main text. These descriptive comparisons show no statistically significant between-group differences in any physical activity outcomes (all p >

0.05), which is unsurprising given the low statistical power and implementation challenges documented throughout the manuscript. We have added a summary statement in the Results section directing readers to these supplementary analyses. We believe this approach appropriately balances transparency (providing the requested data) with scientific rigor (avoiding overinterpretation of underpowered comparisons in the main text). If the reviewer prefers, we can move the most relevant table into the main manuscript, accompanied by a clear note indicating that these analyses are exploratory and underpowered.

Page 10: "*Comparative analyses of the intervention and control groups of participants with complete PA data at the end of the programme (n = 17) are presented in Supplementary Material 2 (Tables S2–S3). As expected given the small sample size and implementation challenges, no statistically significant differences were observed between the groups for any outcome (all p > 0.05). However, we did observe a statistically significant difference in global perceived physical health ($M_{intervention}$ = 2.50, SD = 0.71 vs. $M_{control}$ = 3.71, SD = 1.11, p = .015, scale: 1–5). However, these analyses should be interpreted with caution due to substantial attrition and the study's limited statistical power.*"

Supplementary Material 2. Comparison Between the Intervention and the Placebo Groups
Table S2. Baseline Characteristics of Participants with Complete Post-Intervention Physical Activity Data (N = 17)

***N* = 17**	**Intervention *N* = 10**	**Missing data**	**Placebo *N* = 7**	**Missing data**	
	M (SD)	*N*	*M (SD)*	*N*	*P* value*
Demographics and anthropometrics					
Sex (*n*, %)				1	
Female	4 (40%)	0	1 (14%)	0	
Male	6 (60%)	0	6 (86%)	0	.338
Age (years, *n*, %)					.815
<50	1 (10%)	0	1 (14%)	0	
50–59	4 (40%)	0	2 (29%)	0	
60–69	4 (40 %)	0	2 (29%)	0	
70–79	4 (40%)	0	2 (29%)	0	
>80	0 (0%)	0	0 (0%)	0	
Body mass index (Kg/m^2; *n*, %)					.079
Underweight <18.5	0 (0%)	0	0 (0%)	0	
Normal 18.5–<25	5 (50%)	0	5 (71%)	0	
Overweight 25–<30	4 (40%)	0	2 (29%)	0	
Obese ≥ 30	1 (10%)	0	0 (0%)	0	
Usual level of PA (*n*, %)					.50
Physically inactive	2 (20%)	0	0 (0%)	0	
Some PA	4 (40%)	0	5 (71%)	0	
Regular PA	4 (40%)	0	2 (29%)	0	
High training	0 (0%)	0	0 (0%)	0	

Emotional health-related variables					
Anxiety [1–5]	1.65 (0.91)	0	1.64 (0.85)	0	.987
Depressive symptoms [1–5]	1.68 (0.86)	0	1.39 (0.48)	0	.445
Fatigue [1–5]	2.27 (0.86)	0	2.10 (1.05)	0	.716
Pain intensity [1–5]	1.90 (1.79)	0	0.86 (1.57)	0	.234
Additional health-related variables					
Number of ADL (*n*, %)					.412
No ADL	10 (100%)	0	6 (86%)	0	
≥1 ADL	0 (0%)	0	1 (14%)	0	
Number of IADL (*n*, %)					.644
No IADL	7 (70%)		4 (57%)		
≥1 IADL	3 (30%)	0	3 (43%)	0	
Mobility [1–5]	3.80 (0.42)	0	4.00 (0.00)	0	.143
Global physical health [1–5]	2.50 (0.71)	0	3.71 (1.11)	0	.015
Global mental health [1–5]	3.60 (1.08)	0	4.29 (0.76)	0	.168
Life satisfaction [1–7]	2.30 (0.82)	0	1.71 (0.49)	0	.114

Continued

Table S2. (*Continued*)

N = 17	Intervention *N* = 10	Missing data	Placebo *N* = 7	Missing data	
	M (SD)	*N*	*M (SD)*	*N*	*P* value*
LVEF (%)	52.50 (12.10)	2	55.00 (9.75)	1	.686
MAP (Watt)	124.67 (63.74)	1	159.86 (45.85)	0	.239
Abdominal circumference (cm)	90.50 (8.85)	0	93.00 (9.31)	0	.583
HbA1c (%)	5.69 (1.01)	3	5.30 (0.34)		.358
Triglycerides (mmol/L)	1.27 (0.45)	1	1.55(0.81)	0	.389
LDL (mmol/L)	2.99 (1.17)	0	3.65 (0.55)	0	.187
Systolic blood pressure (mmHg)	123.40 (11.84)	0	120.86 (15.31)	0	.704
Diastolic blood pressure (mmHg)	72.80 (7.90)	0	67.29 (8.79)	0	.196
Cigarette (pack/year)	4.50 (7.62)	0	5.14 (8.86)	0	.875
Comorbidity (*n*, %)				0	
0	2 (20%)	0	0 (0%)	0	.647
1	4 (40%)	0	4 (57%)	0	
≥ 2	4 (40%)	0	3 (43%)	0	
Indication for enrollment in the outpatient CR (*n*; %)					1.000
Acute coronary syndrome NSTEMI	4 (40%)	0	3 (43%)	0	
Acute coronary syndrome STEMI	2 (20%)	0	2 (28.5%)	0	
Acute coronary syndrome UA	1 (10%)	0	2 (28.5%)	0	
Coronary artery bypass graft surgery	0 (0%)	0	0 (0%)	0	
Coronary artery disease without ACS or surgery	1 (10%)	0	0 (0%)	0	
Heart failure	0 (0%)	0	0 (0%)	0	
Valvular surgery	2 (20%)	0	0 (0%)	0	

Motivational variables towards PA					
Perceived capabilities [1–5]	4.40 (0.70)	0	4.71 (0.49)	0	.340
Intention [1–10]	5.65 (1.13)	0	4.86 (2.12)	0	.331
Instrumental attitudes [1–10]	9.10 (1.73)	0	9.71 (0.76)	0	.395
Affective attitudes [1–10]	8.90 (1.73)	0	9.00 (1.16)	0	.896
Approach toward PA	-20.20 (87.36)	3	26.41 (112.71)	0	.404
Approach toward SB	-37.836 (107.02)	3	41.67 (85.07)	0	.150

Abbreviations: M, mean; SD, standard deviation; PA, physical activity; ADL, limitations in activities of daily living; IADL, limitations in instrumental activities of daily living; CR; cardiac rehabilitation; NSTEMI, non-ST elevation myocardial infarction; STEMI, ST elevation myocardial infarction; UA, unstable angina; ACS, acute coronary syndrome; LVEF, left ventricular ejection fraction; MAP, maximal aerobic power; HbA1c, fasting glycated hemoglobin; LDL, low-density lipoprotein-cholesterol; SB, sedentary behaviors. Usual level of PA was assessed with the Saltin-Grimby PA Level Scale. *P-values from independent samples t-tests (numerical variables) or Fisher's exact test (categorical variables). These analyses are severely underpowered (total n = 17) and should be interpreted only as descriptive information. The study experienced 75% attrition and substantial implementation challenges. These results do not provide meaningful evidence regarding intervention effectiveness.

Table S3. Accelerometer-Measured Physical Activity One Week After Discharge from the Programme: Between-Group Comparisons (N = 17)

***N* = 17**	**Intervention *N* = 10**	**Missing data**	**Placebo *N* = 7**	**Missing data**	
	M (SD)	*N*	*M (SD)*	*N*	*P* value*
Daily MVPA (min/day)	60.43 (43.56)	0	59.91 (19.52)	0	.977
Daily sedentary behaviour (min/day)	554.16 (72.03)	0	540.89 (35.59)	0	.661

Abbreviations: M, mean; MVPA: moderate-to-vigorous physical activity. * P-values from independent samples t-tests

2, PA change from baseline to after intervention should be shown in the Table, even for small data.

Response: Thank you for this important suggestion. We agree that comparing physical activity change from baseline to post-intervention would be valuable. Unfortunately, this direct comparison is not possible due to a methodological limitation: baseline physical activity was assessed using the Saltin–Grimby Physical Activity Level Scale (Grimby et al., 2015; https://doi.org/10.1111/sms.12611), which classifies activity into four ordinal categories (physically inactive / some physical activity / regular physical activity / high physical training), whereas post-intervention physical activity was measured via

accelerometer (continuous data). These fundamentally different measurement scales prevent calculation of within-person change scores.

To partially address this limitation, we have compared participants with complete accelerometer data at programme end (n = 17) on both: (1) baseline characteristics, including baseline Saltin-Grimby physical activity categories (Supplementary Table S2), and (2) post-intervention accelerometer-measured physical activity outcomes (Supplementary Table S3). These comparisons show no significant differences at baseline (self-reported physical activity levels, $p = .50$) and at the end of the programme (all accelerometer outcomes $p > .05$), providing descriptive evidence of no differential intervention effect.

3. In the Discussion, a comparison with other studies is recommended. The manuscript briefly notes attrition rates in CBM interventions for other health behaviours. This section could be expanded slightly to discuss whether the observed rates are typical for cardiac rehabilitation interventions more broadly, beyond CBM.

Response: Thank you for this valuable suggestion. We have expanded the Discussion to provide a more comprehensive comparison of our attrition findings with both CBM interventions literature and cardiac rehabilitation programmes more broadly. Relevant citations have been added to support these comparisons (see Discussion, p.15). The revised section situates our attrition rate of 75% within the broader cardiac rehabilitation literature, where dropout rates range from 12–82%.[46–49] It discusses patient characteristics associated with higher attrition in cardiac rehabilitation and proposes that our challenges may reflect the compounding effects of implementing a demanding digital intervention within an already challenging rehabilitation context. This contextualisation emphasises that engagement is a challenge that requires solutions at multiple levels, including intervention design, patient-level factors, and service delivery.

Pages 16–17: "*In the broader context of cardiac rehabilitation programmes, attrition is also a pervasive challenge. Systematic reviews report dropout rates ranging from 12% to 82% across international cardiac rehabilitation programmes.*[46-49] *with substantial heterogeneity attributable to differences in programme design, healthcare system factors, and patient characteristics. Higher attrition rates are consistently observed among older patients, women, individuals with multiple comorbidities, and those from underserved racial, social and ethnic minority groups.*[46-49] *With an observed attrition rate of 75%, our study falls at the higher end of both CBM intervention studies (43–90%) and cardiac rehabilitation*

programmes (12–82%). This suggests that the challenges we encountered may be due to the combination of two context-specific difficulties: implementing a repetitive, cognitively demanding digital intervention in a rehabilitation setting, where sustained engagement is already challenging. Framing our findings within this broader literature highlights that acceptance and adherence are key implementation issues for both digital behaviour-change interventions and standard cardiac rehabilitation services."

VERSION 2 - REVIEW

**Reviewer**	**1**
**Name**	**Rechcinski, Tomasz**
**Affiliation**	**Medical University of Lodz**
**Date**	**19-Oct-2025**
**COI**	

Thank you for modifications of the text after receiving reviewer's comments.

**Reviewer**	**2**
**Name**	**Hirashiki, Akihiro**
**Affiliation**	**Natinal Center for Geriatrics and Gerontology, Cardiology**
**Date**	**17-Oct-2025**
**COI**	

There are no further comments.

**Reviewer**	**3**
**Name**	**O'Cathain, Alicia**
**Affiliation**	**University of Sheffield, Sheffield Centre for Health and Related Research**
**Date**	**30-Oct-2025**
**COI**	**None**

Overview

It is useful to reflect on the failure of RCTs.

This is a challenging paper because it is based partly on quantitative data collected as part of the RCT and partly on feedback from research assistants (RAs) who made notes while delivering the intervention to patients. This latter component is not qualitative research. Does this mean that the research is not useful? I think the research is useful but I am concerned about two things. First, the lack of information about the feedback data. Was all of it collected during the intervention delivery or was some of it collected by speaking to RAs after the RCT was ended? Where did the views of clinical staff come from? There is a quote from medical staff but did an RA write this down during the RCT? The authors are very explicit about the limitations of the data but I think the limitations are made worse by the lack of description of what data is available, where it was collected, and how it was collated and analysed etc. Currently the research is not reproducible. Second, the paper sits uncomfortably between a team's reflections on what went wrong (e.g. the team did not include RAs in the research team) and what was documented in notes made by the RAs (which you might call data). For example, in the research constraints section in the results there are team reflections rather than data-based findings. These issues can be fixed. The research will still have considerable limitations but the authors have been explicit about these limitations throughout the paper.

In the UK it is standard practice now to use qualitative research alongside RCTs of complex interventions like this. A recommendation of this paper could be that there should be planned qualitative research that follows the MRC guidance on the development and evaluation of complex interventions (Skivington et al 2021) for interventions like this in the future. Prior to the RCT some qual research with patients might have identified all these problems with the intervention.

Specific comments

Abstract

What is an acceptance evaluation? It is not a common term and it is best not to use it here.

I do not think you can call this 'patients verbal reports' when the data you have are based on notes made by an RA.

The conclusion should be rewritten to document the learning from the study.

Strengths and limitations section

I am concerned about the use of the term 'qualitative data' when qualitative research was not actually undertaken. Also, the third bullet point should make it clear that RAs made notes during intervention delivery (or did they recollect issues when the RCT was ended?).

PPI

It's a shame that PPI members were not involved because they may have pointed out these problems before the RCT was designed. It is worth having a recommendation in the

discussion that PPI in the design of interventions and RCTs would be useful for future studies.

Major comments on the body of the paper

The main issue is how these feedback data were collected. Did RAs make notes on a questionnaire so are these like open comments on a survey? Did it also rely on RAs reflecting back on what happened after the end of the RCT? How did this occur – did they write things down to share with you? How many offered data? How much data was there? How were data analysed? How did the quotes from medical staff on p12 come about? - did an RA write this down during data collection?

Quotes need to have context – it is usual to report the type of patient who said it and an ID number so people can see that it's not just the same person quoted all the time.

There is a sense that the team are generally happy with this intervention and might make some changes to it in the future. Please give some thought to the potential that this intervention should not be pursued at all in the future.

Minor issues

P4 l54 be clear – is it the report of the acceptability of the trial, the intervention, or both?

P4 l56 propose, not proposed

P5 l38 incomplete sentence

P5 l38 'it' should be 'the intervention'

P7 It is problematic that the patient feedback relies on RA recall with nothing recorded at all so it cannot be called qualitative research

P12 l54 constraints not constrains

p14 l45 a point is made that could be in the results about medical staff reporting that 10% of patients were accompanied by a family member. Does this belong in the results?

P19 please make a subheading of 'strengths and limitations'

The footnote about the different meanings of acceptability and acceptance needs to be in the background of the paper. I had not come across this difference before and had originally wanted the authors to use the word acceptability. It is useful for readers to know that this word is used deliberately.

There is ethics approval for the RCT but is there ethics approval to use the notes made by the RAs?

VERSION 2 - AUTHOR RESPONSE

Reviewer: 1

Dr. Tomasz Rechcinski, Medical University of Lodz

Comments to the Author:

Thank you for modifications of the text after receiving reviewer's comments.

Response: We would like to thank Dr Rechcinski for his/her positive feedback and for acknowledging the modifications we made in response to his/her comments. We are pleased that our revisions have addressed his/her concerns, and we are grateful for the time and expertise he/she dedicated to reviewing our manuscript.

Reviewer: 2
Dr. Akihiro Hirashiki, Natinal Center for Geriatrics and Gerontology
Comments to the Author:
There are no further comments.
Response: We would like to thank Dr Hirashiki for his/her positive feedback and confirmation that no further comments are required. His/her input has been invaluable in helping us to improve our work, and we are glad that our responses met his/her expectations.

Reviewer: 3
Prof. Alicia O'Cathain, University of Sheffield
Comments to the Author:
R3.1. Overview
It is useful to reflect on the failure of RCTs.
This is a challenging paper because it is based partly on quantitative data collected as part of the RCT and partly on feedback from research assistants (RAs) who made notes while delivering the intervention to patients. This latter component is not qualitative research. Does this mean that the research is not useful? I think the research is useful but I am concerned about two things. First, the lack of information about the feedback data. Was all of it collected during the intervention delivery or was some of it collected by speaking to RAs after the RCT was ended? Where did the views of clinical staff come from? There is a quote from medical staff but did an RA write this down during the RCT? The authors are very explicit about the limitations of the data but I think the limitations are made worse by the lack of description of what data is available, where it was collected, and how it was collated and analysed etc. Currently the research is not reproducible. Second, the paper sits uncomfortably between a team's reflections on what went wrong (e.g. the team did not include RAs in the research team) and what was documented in notes made by the RAs (which you might call data). For example, in the research constraints section in the results there are team reflections rather than data-based findings. These issues can be fixed. The research will still have considerable

limitations but the authors have been explicit about these limitations throughout the paper.
In the UK it is standard practice now to use qualitative research alongside RCTs of complex interventions like this. A recommendation of this paper could be that there should be planned qualitative research that follows the MRC guidance on the development and evaluation of complex interventions (Skivington et al 2021) for interventions like this in the future. Prior to the RCT some qual research with patients might have identified all these problems with the intervention.
Response: We would like to thank Dr O'Cathain for his/her thoughtful and constructive feedback, which has helped us to considerably strengthen the clarity and methodological positioning of our manuscript. We agree that the notes provided by research assistants (RAs) should not be described as qualitative data, and we also agree that it is important to avoid using the term "qualitative" in this context. Specifically, feedback data were collected exclusively through verbal reports provided by RAs to the experimental team during the intervention period. The three RAs involved in delivering the intervention shared observations immediately after noting relevant interactions with patients or medical staff – for example, "A patient mentioned that…" or "A member of the medical staff commented that…" These reports were not obtained via structured questionnaires, surveys or direct recordings of participants or staff; instead, they relied on the RAs' recollections of specific interactions at the time they occurred. We have revised the manuscript accordingly, ensuring this distinction is explicit throughout.

Page 2: "***Primary and secondary outcome measures.*** *Acceptance was assessed using behavioural (e.g., enrolment and engagement rates), cognitive (e.g., perceived effectiveness), and emotional (e.g., affective evaluation) indicators. The cognitive and emotional indicators were derived from verbal feedback documented by the research assistants (RAs) based on patients' reactions during the intervention period. These observations do not constitute qualitative research as defined by methodological standards; they were informal notes provided by RAs during intervention delivery and were not collected or analysed using qualitative research methods."*

Page 3: "***Strengths and limitations of this study***

- *This study uses a multidimensional assessment of acceptance by examining behavioural, cognitive, and emotional indicators of a cognitive-bias modification intervention.*

- *Including informal verbal feedback from research assistants (RAs) offer valuable insights into patients' experience and perception of the intervention in a real-world cardiac rehabilitation setting.*
- *Acceptance was assessed primarily through RAs' verbal feedback collected during intervention delivery, rather than direct patients' reports or standardised measures, which may introduce recall bias and limit the reliability of the findings.*"

We also agree on the need for a clearer description of the RAs' notes, including details of the information collected, how and when it was collected, and the form it was collected in. We now specify that all RA notes were collected directly during the delivery of the intervention and clarify how they were recorded, collated and interpreted. We also explain that the views of clinical staff were captured through comments documented in real time by the RAs. We also acknowledge the reviewer's point that the manuscript previously sat between team reflections and data-based findings. In the revised version, we now clarified this distinction by separating reflective elements from material derived from RA documentation. We hope this improves transparency and enhances reproducibility within the limits of the available data.

> **Pages 7–8:** "*Feedback reports were compiled by research assistants during the intervention based on verbal reports from patients and medical staff, which were not recorded or documented at the time. These reports were not formally recorded or documented, nor were they obtained through structured methods such as questionnaires, surveys, or direct recordings. Instead, they consisted of unsystematic observations, including patients' reactions, views of clinical staff, practical difficulties with the protocol and implementation barriers. These were*

verbally relayed by research assistants immediately after relevant interactions. Due to their informal and unstructured nature, these observations do not adhere to the methodological standards of qualitative research. Instead, they served as contextual documentation that emerged organically during the intervention's delivery. We analyse them here as supplementary contextual information to better understand implementation barriers, while explicitly acknowledging their methodological limitations. This reliance on research assistants' recollections of patients' verbal feedback may introduce recall bias, thereby limiting the validity and accuracy of these indicators."

Pages 9–10: "*During the intervention period, research assistants documented any spontaneous patients' and medical staff feedback related to perceived effectiveness of the intervention, with particular attention to verbal evaluations of the approach-avoidance training task.*

Burden. Prior and during the intervention period, the burden of the intervention was assessed using patients' and medical staff spontaneous feedback. Research assistants verbally reported any such feedback relating to the perceived effort required to participate in the intervention to the research team, with particular attention to verbal evaluations of the task perceived difficulty or demands. However, this feedback was not (formally) recorded or documented by the research assistants."

Emotional Indicators

Attitudes Towards the Intervention. Prior and during the intervention period, affective attitudes towards the intervention (i.e., the extent to which participants perceived the intervention as something unpleasant or pleasant, boring or fun) were assessed using patients' spontaneous feedback. Research assistants verbally

reported any spontaneous patient feedback related to their feelings about the intervention to the research team, with particular attention to verbal ratings of the level of boredom-fun and disinterest-interest towards the CBM task. This feedback was not (formally) recorded or documented by the research assistants.”

Pages 12–13: “***Cognitive Indicators***

Prior to the start of the intervention period, patients demonstrated a moderate perceived effectiveness of the intervention (M = 3.09 on a scale ranging from 1 to 5; Supplementary Material 4, Table S4 and Figure S2). Informal feedback from research assistants indicated that some patients were sceptical about the effectiveness of the intervention. For instance, patients questioned how the task could help them become more active. Some were also reluctant to participate in activities they perceived as unnecessary, stating that they did not want to take on extra tasks during their rehabilitation. This reluctance was probably because of the demanding nature of the rehabilitation protocol, which was part of their standard clinical care (see Supplementary Material 5 for details).

Another factor that may have reinforced the perception of the intervention as an additional burden was the lack of integration of research assistants into the healthcare team. Despite our efforts to embed them within the clinical setting, research assistants were not fully integrated (e.g., attending weekly team seminars or receiving formal introductions as part of the clinical staff). This separation likely contributed to the intervention being viewed as an extra task, distinct from the regular rehabilitation protocol, thereby increasing the perceived burden. This separation likely increased the perceived burden not only for patients but also for healthcare professionals, who may have considered it an

extra task outside their standard clinical responsibilities. Additionally, informal feedback from research assistants suggests that medical staff, including doctors and physiotherapists, faced challenges in reminding patients to attend their scheduled CBM sessions. Staff reported that time constraints and competing priorities within the rehabilitation protocol hindered their ability to consistently prompt patients to participate in these sessions."

Page 13: "***Emotional Indicators***

Additional feedback from research assistants highlighted significant issues related to patients' boredom and disinterest. Patients reportedly found the CBM sessions monotonous and perceived them as long and unengaging. They also showed little interest in interacting with tablet-based activities. Furthermore, patients criticised the visual presentation of the CBM task, commenting that the images were unappealing and suggesting that a more colourful design might improve their experience. As the intervention progressed, boredom seemed to increase as feedback about monotony became more frequent. It is possible that the novelty of the intervention, a key factor in maintaining interest and curiosity,[38] *wore off over time, contributing to the increased feelings of boredom.*"

Page 14: "*However, these results should be interpreted in the context of the informal, unplanned and unsystematic data collection process, which relied on the research assistants' recollections of their interactions with patients.*"

Page 21: "*This retrospective study has several important limitations. First, the data analysed were not collected systematically with the purpose of evaluating acceptance. Rather, they emerged from informal observations of the implementation process relayed by research assistants during the original trial.*"

We also appreciate the suggestion to emphasise the value of planned qualitative research alongside RCT of complex interventions. We agree that this approach is important, and we now refer explicitly to Skivington et al. (2021) to highlight the relevance of the Medical Research Council (MRC) framework. We acknowledge that planned qualitative work conducted prior to the RCT could have revealed many of the implementation issues we encountered.

> **Page 20:** "*However, it is also crucial to ensure that participants, regardless of their age, socio-economic background, or health conditions, possess adequate skills to engage with digital interventions.*[64] *Addressing this challenge requires a systematic and participatory approach, as outlined in the Medical Research Council (MRC) Framework for developing and evaluating complex interventions.*[65] *According to this framework, successful interventions must be developed through iterative phases that prioritise not only efficacy, but also contextual factors (e.g. alignment with participants' routines, cultural norms and resource availability), stakeholder engagement and adaptability. Therefore, complex interventions, particularly those involving digital tools, must be developed with a deep understanding of the contextual factors influencing their acceptance and feasibility. To address this challenge, the co-production of the protocol by patients, health professionals, researchers, and PPI members, alongside conducting pilot studies prior to full implementation, can help ensure that interventions are accessible, engaging, and effective for the target population.*[65-67] *For instance, qualitative research conducted during the feasibility phase may reveal disparities in digital literacy, varying levels of technological proficiency, or cultural preferences that could impact engagement.*

Adopting the MRC approach, which includes development, feasibility testing, evaluation and implementation, enables future research to systematically integrate stakeholder feedback and contextual adaptations. This approach ensures that digital interventions are technically accessible, culturally appropriate, and feasible for all participants, thereby enhancing their acceptance and impact.

Implementing these recommendations would improve the quality of research, but careful planning and resource allocation are required. Therefore, researchers should explicitly outline these steps in grant proposals and justify the need for additional time, funding and personnel to conduct qualitative research, pilot studies and stakeholder engagement. Although these measures would extend trial timelines and increase costs, they are essential for maximising the rigour, relevance and impact of complex interventions. Grant institutions should invest in these processes, recognising that they will yield higher-quality, more informative research and ultimately deliver greater value for public and scientific benefit."

Table 1. Potential Reasons for Failure and Recommendations for Future Studies

Difficulty with patient acceptance	Patients perceived the task as useless and boring.	Enhance the perceived value of the task by linking it clearly to PA behaviour and reducing its monotony.
	Task was lengthy, monotonous, repetitive and of low subjective value.	Including animations and "vitality gauges" that can increase or decrease depending on the action (e.g., approaching or avoiding PA).
	Lack of perceived connection between the task and its impact on PA behaviour.	Integrate more engaging and intrinsically motivating tasks, such as gamification or consequence-based interventions (e.g., ABC training[27]).
	Scepticism about the effectiveness of digital interventions.	Employ emerging technologies like virtual reality to create more appealing interventions.
		Co-production of protocol and pilot study.
		MRC-based qualitative research.

Abbreviations: ABC, Antecedents Behaviours Consequences; MRC, Medical Research Council; PA, physical activity; PPI, patient and public involvement.

Specific comments

R3.2. Abstract

What is an acceptance evaluation? It is not a common term and it is best not to use it here.

Response: We thank the reviewer for pointing this out. We agree that the term "acceptance evaluation" is uncommon and may create ambiguity. We have therefore removed it from the "Design" section.

Page 2: "***Design.*** *Retrospective evaluation of a pre-registered clinical trial.*"

R3.3. I do not think you can call this 'patients verbal reports' when the data you have are based on notes made by an RA.

Response: We thank the reviewer for raising this important point. We agree that these data should not be described as "patients' verbal reports", since they consist of informal verbal feedback from research assistants on patients' general reactions to the intervention, rather

than direct patient accounts. We have revised the abstract to ensure this distinction is clear and to avoid any ambiguity.

> **Page 2:** "***Primary and secondary outcome measures.*** *Acceptance was assessed using behavioural (e.g., enrolment and engagement rates), cognitive (e.g., perceived effectiveness), and emotional (e.g., affective evaluation) indicators. The cognitive and emotional indicators were derived from verbal feedback documented by the research assistants (RAs) based on patients' reactions during the intervention period. These observations do not constitute qualitative research as defined by methodological standards; they were informal notes provided by RAs during intervention delivery and were not collected or analysed using qualitative research methods."*

R3.4. The conclusion should be rewritten to document the learning from the study.

Response: We thank the reviewer for this suggestion. We agree that the conclusion should explicitly highlight the key lessons learned from the study. We have therefore rewritten it to emphasise the key barriers identified, as well as the methodological and practical lessons that can inform the development and evaluation of future interventions.

> **Page 3:** "***Conclusion.*** *The low engagement and acceptance observed in the IMPACT trial reveal highlight several key barriers, such as perceived task irrelevance, task monotony, and task boredom, that undermine the acceptance and feasibility of this digital CBM intervention in cardiac rehabilitation. These findings emphasise the importance of designing patient-centred interventions, ensuring their seamless integration into clinical contexts, and conducting qualitative research prior to implementation to anticipate potential barriers."*

R3.5. Strengths and limitations section

I am concerned about the use of the term 'qualitative data' when qualitative research was not actually undertaken.

Also, the third bullet point should make it clear that RAs made notes during intervention delivery (or did they recollect issues when the RCT was ended?).

Response: We thank the reviewer for highlighting these important points. We fully agree that the term "qualitative data" is inappropriate in this context, and it has now been removed from the manuscript. We have also revised the bullet point to clarify that the feedback was collected by RAs while the intervention was being delivered, rather than afterwards or from the participants directly. This distinction has been made to improve transparency regarding data collection and ensure the source of the information is accurately reflected.

> **Page 2:** "***Primary and secondary outcome measures.*** *Acceptance was assessed using behavioural (e.g., enrolment and engagement rates), cognitive (e.g., perceived effectiveness), and emotional (e.g., affective evaluation) indicators. The cognitive and emotional indicators were derived from verbal feedback documented by the research assistants (RAs) based on patients' reactions during the intervention period. These observations do not constitute qualitative research as defined by methodological standards; they were informal notes provided by RAs during intervention delivery and were not collected or analysed using qualitative research methods."*
>
> **Page 3:** "***Strengths and limitations of this study***
>
> - *This study uses a multidimensional assessment of acceptance by examining behavioural, cognitive, and emotional indicators of a cognitive-bias modification intervention.*

- *Including informal verbal feedback from research assistants (RAs) offer valuable insights into patients' experience and perception of the intervention in a real-world cardiac rehabilitation setting.*
- *Acceptance was assessed primarily through RAs' verbal feedback collected during intervention delivery, rather than direct patients' reports or standardised measures, which may introduce recall bias and limit the reliability of the findings.*"

R3.5. PPI

It's a shame that PPI members were not involved because they may have pointed out these problems before the RCT was designed. It is worth having a recommendation in the discussion that PPI in the design of interventions and RCTs would be useful for future studies.

Response: We thank the reviewer for highlighting the importance of patient and public involvement (PPI) in the design of RCTs. We fully agree that PPI members could have provided valuable insights into the hospital environment and implementation challenges, potentially improving the design and feasibility of the intervention. Drawing on the broader literature, including the conceptual review by Fredriksson et al. (2025, https://doi.org/10.1016/j.ssmqr.2024.100517), which underscores the impact of PPI on individuals, health systems, and decision-making, we have added a recommendation to the discussion section. This recommendation emphasises the importance of integrating PPI into the early stages of intervention and trial design to improve the acceptability and feasibility of future studies.

Pages 15–16: "*Additionally, early and meaningful patient and public involvement (PPI) in the design and planning of interventions can provide critical insights*

into potential barriers and facilitators. PPI members help to ensure that interventions are patient-centred, feasible and acceptable by offering perspectives on the hospital environment, patient preferences and practical challenges that researchers might otherwise overlook.[43,44] *For instance, their input can inform the development of materials and the integration of the protocol into patients' routines, reducing perceived burden and increasing engagement.*"

Page 21: "*To address this challenge, the co-production of the protocol by patients, health professionals, researchers, and PPI members, alongside conducting pilot studies prior to full implementation, can help ensure that interventions are accessible, engaging, and effective for the target population.*[65,66]"

Table 1. Potential Reasons for Failure and Recommendations for Future Studies

Issue	Reasons	Recommendations
Difficulty with patient enrolment	Protocol perceived as separate from care, high commitment burden.	Integrate the protocol as a central part of rehabilitation.
		Family support.
		Involvement of PPI in the design of the intervention
		Explore low-friction, automated interventions that minimise participant burden (e.g., Heartphone app[25]).
	Research assistants not fully integrated in the hospital unit.	Ensure research assistants are fully embedded in the healthcare team.
	Inconsistent research assistant availability.	Align training sessions with patient schedules to minimise perceived burden.
	Logistical issues with tablets.	Ensure regular maintenance for all equipment.

R3.6. Major comments on the body of the paper

The main issue is how these feedback data were collected. Did RAs make notes on a questionnaire so are these like open comments on a survey? Did it also rely on RAs reflecting back on what happened after the end of the RCT? How did this occur – did

they write things down to share with you? How many offered data? How much data was there? How were data analysed? How did the quotes from medical staff on p12 come about? - did an RA write this down during data collection?

Quotes need to have context – it is usual to report the type of patient who said it and an ID number so people can see that it's not just the same person quoted all the time.

<u>Response:</u> We would like to thank the reviewer for raising these important questions about how feedback data was collected and used in our study. We acknowledge the need for clarity on this point. As previously described, feedback data were collected exclusively through verbal reports provided by RAs to the experimental team during the intervention period. Specifically, the three RAs involved in delivering the intervention shared observations immediately after noting relevant interactions with patients or medical staff – for example, "A patient mentioned that…" or "A member of the medical staff commented that…" These reports were not obtained via structured questionnaires, surveys or direct recordings of participants or staff; instead, they relied on the RAs' recollections of specific interactions at the time they occurred.

We also agree with the reviewer that presenting these reports as direct quotes could be misleading. This method of data collection introduces potential biases, including recall bias and the risk of misinterpretation, which is a notable limitation. To address this issue, we have revised the manuscript so that these observations are clearly described as informal feedback from RAs, rather than as direct quotations from participants or staff. We have also emphasised the limitations of this approach, including the lack of standardised documentation, and removed any presentation of these reports as verbatim quotes. These revisions aim to improve transparency and ensure that readers understand the nature and constraints of the data.

Pages 7–8: "*Feedback reports were compiled by research assistants during the intervention based on verbal reports from patients and medical staff, which were not recorded or documented at the time. These reports were not formally recorded or documented, nor were they obtained through structured methods such as questionnaires, surveys, or direct recordings. Instead, they consisted of unsystematic observations, including patients' reactions, views of clinical staff, practical difficulties with the protocol and implementation barriers. These were verbally relayed by research assistants immediately after relevant interactions. Due to their informal and unstructured nature, these observations do not adhere to the methodological standards of qualitative research. Instead, they served as contextual documentation that emerged organically during the intervention's delivery. We analyse them here as supplementary contextual information to better understand implementation barriers, while explicitly acknowledging their methodological limitations. This reliance on research assistants' recollections of patients' verbal feedback may introduce recall bias, thereby limiting the validity and accuracy of these indicators.*"

Pages 9–10: "*During the intervention period, research assistants documented any spontaneous patients' and medical staff feedback related to perceived effectiveness of the intervention, with particular attention to verbal evaluations of the approach-avoidance training task.*

Burden. Prior and during the intervention period, the burden of the intervention was assessed using patients' and medical staff spontaneous feedback. Research assistants verbally reported any such feedback relating to the perceived effort required to participate in the intervention to the research team, with particular attention to verbal evaluations of the task perceived

difficulty or demands. However, this feedback was not (formally) recorded or documented by the research assistants."

Emotional Indicators

Attitudes Towards the Intervention. Prior and during the intervention period, affective attitudes towards the intervention (i.e., the extent to which participants perceived the intervention as something unpleasant or pleasant, boring or fun) were assessed using patients' spontaneous feedback. Research assistants verbally reported any spontaneous patient feedback related to their feelings about the intervention to the research team, with particular attention to verbal ratings of the level of boredom-fun and disinterest-interest towards the CBM task. This feedback was not (formally) recorded or documented by the research assistants."

Pages 12–13: "*Cognitive Indicators*

Prior to the start of the intervention period, patients demonstrated a moderate perceived effectiveness of the intervention (M = 3.09 on a scale ranging from 1 to 5; Supplementary Material 4, Table S4 and Figure S2). Informal feedback from research assistants indicated that some patients were sceptical about the effectiveness of the intervention. For instance, patients questioned how the task could help them become more active. Some were also reluctant to participate in activities they perceived as unnecessary, stating that they did not want to take on extra tasks during their rehabilitation. This reluctance was probably because of the demanding nature of the rehabilitation protocol, which was part of their standard clinical care (see Supplementary Material 5 for details).

Another factor that may have reinforced the perception of the intervention as an additional burden was the lack of integration of research assistants into the

healthcare team. Despite our efforts to embed them within the clinical setting, research assistants were not fully integrated (e.g., attending weekly team seminars or receiving formal introductions as part of the clinical staff). This separation likely contributed to the intervention being viewed as an extra task, distinct from the regular rehabilitation protocol, thereby increasing the perceived burden. This separation likely increased the perceived burden not only for patients but also for healthcare professionals, who may have considered it an extra task outside their standard clinical responsibilities. Additionally, informal feedback from research assistants suggests that medical staff, including doctors and physiotherapists, faced challenges in reminding patients to attend their scheduled CBM sessions. Staff reported that time constraints and competing priorities within the rehabilitation protocol hindered their ability to consistently prompt patients to participate in these sessions."

Page 13: "*Emotional Indicators*

Additional feedback from research assistants highlighted significant issues related to patients' boredom and disinterest. Patients reportedly found the CBM sessions monotonous and perceived them as long and unengaging. They also showed little interest in interacting with tablet-based activities. Furthermore, patients criticised the visual presentation of the CBM task, commenting that the images were unappealing and suggesting that a more colourful design might improve their experience. As the intervention progressed, boredom seemed to increase as feedback about monotony became more frequent. It is possible that the novelty of the intervention, a key factor in maintaining interest and curiosity,[38] wore off over time, contributing to the increased feelings of boredom."

Page 14: "*However, these results should be interpreted in the context of the informal, unplanned and unsystematic data collection process, which relied on the research assistants' recollections of their interactions with patients.*"

Page 21: "*This retrospective study has several important limitations. First, the data analysed were not collected systematically with the purpose of evaluating acceptance. Rather, they emerged from informal observations of the implementation process relayed by research assistants during the original trial.*"

R3.7. There is a sense that the team are generally happy with this intervention and might make some changes to it in the future. Please give some thought to the potential that this intervention should not be pursued at all in the future

Response: We thank the reviewer for his/her comment on the future of this intervention. Based on the challenges observed in this study, such as low patient engagement and perceived irrelevance, we have decided not to continue with this specific RCT. This decision has been explicitly stated in the manuscript to avoid any misunderstanding. Although retraining automatic approach-avoidance tendencies remains an interesting adjunct to classical interventions, this implementation was not engaging enough to ensure adherence. Future implementations could incorporate gaming features, such as those used in ABC training procedures, to enhance intrinsic motivation and engagement. In the revised version of the manuscript, we have further strengthened these points.

Page 6: "*The IMPACT trial faced significant challenges, particularly regarding the acceptance of the intervention among patients, which is defined as a posteriori pragmatic evaluation of an activity (e.g., an intervention).*[33] *As a low acceptance significantly hinders the success of an intervention, we deemed important to better understand the reasons underlying the low acceptance of our*

intervention. Given these challenges, the IMPACT trial has since been terminated as it has proven unfeasible in its original form.

Pages 20: "*To further mitigate boredom, it is important to recognise that highly repetitive tasks can be disengaging, even over short periods of time.*[59] *A key lesson learned from this trial is that expecting patients' adherence to a task that does not provide immediate rewarding value is unrealistic. One promising solution is to incorporate more intrinsically motivating tasks. For example, the ABC training task can include animations and "vitality gauges" that can increase or decrease depending on the action (e.g., approaching or avoiding PA).*[29] *The gamified nature of this task enhances its intrinsic value compared to traditional approach-avoidance tasks.*[60,61]"

R3.8. Minor issues

P4 l54 be clear – is it the report of the acceptability of the trial, the intervention, or both?

Response: The main purpose of this article is to report on the acceptance of both the IMPACT trial and the Cognitive Bias Modification intervention. Specifically, we examine the acceptance of the trial's implementation – including patient and medical staff engagement, recruitment challenges, and the overall feasibility of study procedures – as well as RAs verbal feedback of patients' perceptions of the CBM intervention tasks. This has been clearly addressed in the manuscript.

Page 4: "*The main purpose of this article is to report on the acceptance of the IMPACT trial, which was based on a CBM intervention for cardiac rehabilitation patients.*"

P4 l56 propose, not proposed

Response: This has been amended, thank you.

> **Page 4:** "*We then propose strategies to improve effectiveness of future interventions.*"

P5 l38 incomplete sentence

Response: The sentence has been completed, thank you.

> **Page 5:** "*Patients were excluded if they had a medical contraindication to physical activity.*"

P5 l38 'it' should be 'the intervention'

Response: "It" has been replaced by "the intervention", thank you.

> **Page 5:** "*The intervention initially consisted of a training programme of 12 sessions over 3 weeks (i.e., an average of 4 sessions per week) using a Visual Approach/Avoidance by the Self Task (VAAST[32]).*"

P7 It is problematic that the patient feedback relies on RA recall with nothing recorded at all so it cannot be called qualitative research

Response: We thank the reviewer for pointing this issue out and agree with them. The term "qualitative" has been removed from the manuscript.

> **Page 7:** "*As a result, patients' verbal feedback was not included in the initial protocol. (see Cheval et al.[23]).*"

P12 l54 constraints not constrains

Response: This has been amended, thank you.

Page 9: "*Resources Constraints*"

Page 13: "***Resources Constraints***"

R3.9. p14 l45 a point is made that could be in the results about medical staff reporting that 10% of patients were accompanied by a family member. Does this belong in the results?

Response: We thank the reviewer for this comment. We agree that the information regarding family accompaniment is not directly related to the acceptability or engagement outcomes of the intervention, but rather provides descriptive context about the study population. Therefore, it is more appropriate to present this information in the descriptive results section rather than in the results focused on acceptability outcomes.

Page 10: "*Patients also reported low-to-moderate levels of anxiety (M = 1.79, SD = 0.90, scale: 1–5), depressive symptoms (M = 1.62, SD = 0.76, scale: 1–5), fatigue (M = 2.55, SD = 1.06, scale: 1–5), and pain intensity (M = 2.10, SD, 2.29, scale: 1–5) in their daily life. The medical team reported that around 10% of patients were accompanied by a family member during programme sessions.*"

Page 15: "*In addition, the lack of family involvement may have compounded these challenges further. Despite explicit invitations, the medical team reported that only 10% of patients were accompanied by a family member during programme sessions.*"

R3.10. P19 please make a subheading of 'strengths and limitations'

Response: A subheading "Strengths and Limitations" has been added, thank you.

Page 20: "***Strengths and Limitations***"

R3.11. The footnote about the different meanings of acceptability and acceptance needs to be in the background of the paper. I had not come across this difference before and had originally wanted the authors to use the word acceptability. It is useful for readers to know that this word is used deliberately.

Response: The footnote has been moved to the background of the paper, thank you.

> **Page 7:** "*It should be noted that the TFA uses the term "acceptability" to refer to the evaluation of an intervention before, during, and after its implementation.*[34] *However, some authors propose a distinction between the a priori evaluation of a task (acceptability) and the actual use of the task (acceptance).*[33] *In this report we have used only the term "acceptance" to ensure internal consistency and readability. This choice was made because patient feedback was primarily reported during the intervention period, not before."*

R3.11. There is ethics approval for the RCT but is there ethics approval to use the notes made by the RAs?

Response: We thank the reviewer for raising this important question regarding ethical approval. We acknowledge that the informal verbal feedback provided by RAs to the research team was not explicitly included in the original ethics approval for the RCT. However, these observations consisted solely of general, anonymised insights, and were not treated as primary or formal data. They were not systematically recorded or transcribed either, but rather served as contextual information to help the research team understand barriers to engagement and implementation. Importantly, no personal identifiers were used, and the primary outcomes and structured data reported in the manuscript were fully covered by the approved RCT protocol.
